# Response to Comment on: “Sirolimus versus mycophenolate mofetil for the treatment of lupus nephritis: Results from a real-world CSTAR cohort study”

**DOI:** 10.1515/rir-2026-0026

**Published:** 2026-07-13

**Authors:** Wei Bai, Mengtao Li

**Affiliations:** Department of Rheumatology and Clinical Immunology, Peking Union Medical College Hospital, Chinese Academy of Medical Sciences, Peking Union Medical College, Beijing, China

Dear Editor,

We sincerely appreciate Dr. Bakay’s insightful comments on our article “Sirolimus versus mycophenolate mofetil for the treatment of lupus nephritis: Results from a real-world CSTAR cohort study”.^[1]^ Dr. Bakay’s comments regarding methodological aspects, safety profiles, and long-term efficacy are valuable and provide important clues for further research designs. As emphasized in our original article, this real-world study has inherent limitations, and we would like to respond to Dr. Bakay’s comments based on the data from the CSTAR registry, details of our study, and relevant evidence.

First, regarding the issues of small sample size, insufficient statistical power, low renal biopsy rate and high attrition rate raised by Dr. Bakay. These limitations are closely related to the nature of real-world study and the usage of sirolimus in lupus nephritis (LN) treatment. Currently, sirolimus is off-labelly used for treating LN, resulting in a relatively small number of eligible patients in clinical practice. In our study, 63 patients were treated with sirolimus, while the comparator mycophenolate mofetil (MMF), as the standard of care (SoC) for LN, were used by 622 patients. After propensity score matching (PSM) to balance the baseline characteristics, the sample size was reduced to 53 patients per group, which inevitably affected the statistical power of the study. Regarding the low renal biopsy rate (~25% in our study), we fully agree with Dr. Bakay’s suggestion that stratified analyses by International Society of Nephrology/Renal Pathology Society (ISN/RPS) classification or chronicity index would enhance the robustness of conclusions. However, the data in CSTAR registry showed that the renal biopsy rate for treatment-naive LN patients was only 16.5%.^[2]^ Therefore, improving the renal biopsy rate in LN patients is one of the key goals of the construction of systemic lupus erythematosus (SLE) Standardized Diagnosis and Treatment Centers in China.^[3]^ We are actively promoting renal biopsy in clinical practice through multi-center collaboration, and striving to make up this deficiency in subsequent studies. Moreover, the high attrition rate is also a common challenge in real-world studies, mainly due to factors such as loss of follow-up (migration, change of medical institutions), voluntary withdrawal, and disease progression leading to treatment adjustments. Overall, we fully agree with Dr. Bakay that high-quality, randomized, double-blind, controlled clinical trials involving more patients with renal biopsy data and extended follow-up duration are needed to verify the long-term efficacy of sirolimus, and this is exactly the research direction we are focusing on.

Second, for the safety of sirolimus, especially the imbalance in adverse events (AEs) (10 *vs*. 1) and the occurrence of mild renal insufficiency, we reviewed the 10 AEs in the sirolimus group, and all of them were mild, tolerable, and reversible after dose reduction or drug withdrawal. One patient developed alopecia and one developed mild skin rash, however, it could not be ruled out with active lupus. One patient had leukopenia (from 4.6×10^9^/L to 3.2×10^9^/L), which recovered after dose reduction; two patients had mild respiratory tract infections, which improved after antibiotic treatment; one patient had menstrual abnormalities and one had facial edema, both mild and tolerable without stopping the drug. These events are consistent with the safety profile of sirolimus reported in previous literature. Regarding the three cases of mild renal insufficiency in the sirolimus group, the changes in serum creatinine levels and sirolimus treatment duration were as follows: 73→110 μmol/L (sirolimus 1 mg/d for 6 months), 143→156 μmol/L (sirolimus 1 mg/d for 6 months), and 94→97 μmol/L (sirolimus 1 mg/d for 9 months). Renal function stabilized or recovered after drug withdrawal or dose reduction in these patients. Notably, all three patients had a history of calcineurin inhibitor (CNI) use, including two received tacrolimus and one received cyclosporine. Two of them discontinued CNI due to CNI-related nephrotoxicity, and one was treated concurrently with angiotensin receptor blockers (ARBs). This suggests that caution should be exercised when adding sirolimus to patients who are intolerant to CNIs, and attention should be paid to the risk of renal damage when combining sirolimus with other drugs that may increase serum creatinine.

Third, for the treat-to-target (T2T) approach and long-term disease control, we agreed with you that the T2T strategy is crucial for improving the prognosis of LN, and we implemented this strategy based on the European Alliance of Associations for Rheumatology (EULAR) recommendations.^[4]^ As Dr. Bakay pointed out, the long-term durability of sirolimus beyond one year needs to be further confirmed. Currently, we are conducting a randomized controlled trial (RCT) on sirolimus for the treatment of SLE and long-term follow-up of the CSTAR cohort. We are looking forward to providing more reliable evidence on the long-term efficacy of sirolimus through these subsequent studies.

Finally, we would like to thank you again for your interest in our study and your valuable comments, including high-quality RCTs with biopsy-proven patients, adequate sample size, and longer follow-up cohort. Your comments will help us to better recognize the strengths and limitations of our study, and we will take these suggestions into account in our future research to provide more reliable evidence for the clinical management of LN. We look forward to having more discussions with you on this topic.
